# Dental Education—Rejoinder

**Published:** 1884-08

**Authors:** B. Merrill Hopkinson

**Affiliations:** No. 58 Saratoga Street; Baltimore


### REPLY BY DR. HOPKINSON.

The reply to my article on "Dental Education," I should have termed it "Lack of Dental Education," published in the July number of the INDEPENDENT PRACTITIONER, calls for a rejoinder, and I will do myself the honor to answer the personal attacks of my illustrious confreres, nothing else being required. I will say a few words, which will, I think, successfully clear away the fog from the minds of my distinguished brothers, and at the same time place myself in a better light before the profession of which I have the honor to be a member, having been placed in an unwarrantably false light by the faculty of the B. C. D. S. There are many things in the able reply to my modest paper, which I confess fill me with just choler, but as I believe I am generally considered to be a gentleman, suffering much, especially for the benefit of the brethren, and of a modicum of goodness, I will smother the feelings of wrath that are swelling up, and in reply use sledge-hammer blows from the pen to wipe out the slurs of men who distrust my motives, given to the profession in simple and straightforward English.

I was truly surprised to read in the reply, after my most honest statement of my regret for feeling myself obliged to write of the irregularities known to me, regarding it in the light of pure professional duty, that I was rated as only the mouthpiece by which the faculty of the University of Maryland spoke, and in opposition to the B. C. D. S. This is the first time in a somewhat varied experience in life, that my motives have been doubted when I have made a positive statement, leading persons naturally to judge me contrariwise, and this, together with the personal animus of the reply, shows a desire to evade the important charges by making personal attacks upon myself and others, and by trying to show that good and pure motives are not what they seem, but are cloaks to cover a feeling of rivalry and prejudice. Once again let me assure the professional world that my article was written without fear or favor of any man, or set of men, and I would have it distinctly understood by the "doubting Thomas" of the B. C. D. S., as well as by all members of my profession, that I wrote the article from a perfectly independent standpoint, and without the knowledge of any man, save my father.

I think the above statement ought to do away with the feeling that I was aided or abetted in the production of my paper upon "Dental Education" by any member or members of the dental department of the University of Maryland. Now, as regards my official connection with the University of Maryland, and this is a point I wish to emphasize particularly. The catalogue for the dental department for this year contains my name as Assistant Dem. of Operative Dentistry, a very humble but sometimes useful position. My name was placed there under a misapprehension and *entirely*

without my *knowledge* or *consent.* The day I received the catalogue from the Dean I sent him my unconditional resignation, which was of course immediately accepted. My resignation preceded the publication of my article, and indeed the writing of it by quite a lapse of time, therefore, I *could not* have been connected with the University of Maryland, even in the "inferior position" of which my brothers speak so sneeringly. I think this will wipe out the suggestions of men who prefer to use petty slurs in the defense of what they should know and feel to be wrong, instead of defending their cause, weak though it be, in a legitimate and upright manner. Alas for the supposed perspicacity of my illustrious opponents! All the charges I have made against the B. C. D. S. are admitted in toto, and by members of the erring faculty, *as they were made,* so it is not necessary to say more in connection with them. I did not intend in my article to imply that all of the irregularities of the B. C. D. S. were confined to *last year;* far from it, for in the class of '83, at least two other like cases appear, one of them being A. D. Barrett of Virginia, of which case Dr. J. W. Foreman of Norfolk, Va., knows, and writes me about; the other, George Horatio Jones, of England, who received his diploma, so says "Dame Rumor," by proxy, and of course *in absentia.* How is that, did you ask? I pray some one acquainted with the facts, and there must be many, tell us all about it. Dr. Foreman also says that in the year we graduated, viz.: 79-80, two gentlemen received their sheepskins without attendance. It may be so; will he be kind enough to give us full particulars, and let us go to the bottom of this thing and find out the whole truth. The famous "Visitors' Letter," I confess I overlooked, and in it may be found a small hole through which to crawl, and by the amount of crawling done, I should think by this time the hole would admit the passage of a veritable "jumbo" of unlawful evasion. The necessities of a state, when Tom, Dick, and Harry can call themselves "Doctor," and open an office for the purpose of rendering professional services, demand such a "board" to investigate the ability of such pretenders; but the Faculty of a well-regulated and incorporated dental school must be a singular combination of intellects, if they require a committee of a so-called

"visiting board," to advise them what they had better do with students, whom they, the Faculty, have had under their sole charge for a period of months, and know before the days of the dread and awful "final," full well the capabilities of each student in the graduating class. They must indeed be modest men in a high degree, or else they are too anxious to have a means whereby they can evade the letter of the law as contained in their requirements for graduation. The visitors, better call them "helps to graduation," were indeed a boon in the cases of which I have written. That same "board of visitors," as well as several members of the present faculty of the B. C. D. S., assisted in the decay, and were present at the death of the great and famous "Maryland Dental College," which passed away at the tender age of six years! R. I. P.!!

Verily, verily, I say unto the governing faculty of the B. C. D. S., I am the same B. M. Hopkinson who signed the paper in question, and let me add *for cause*, and I have never been able to see any reason why I should have done otherwise, and my sole regret has been ever since that the faculty did not appear to have as good judgment as the petitioning students.

Verily, verily, I say again, *anonymous affidavits*, like anonymous letters, are "not brave things" for gentlemen of age, wisdom and erudition, and I would suggest leaving them to the young, foolish and untutored. As for the suggestion on page 366 of the reply, concerning the fees which "slipped through the hands" of the Professors of the University of Maryland, I do not esteem it worthy of notice, and it must have been the coinage of the brain of some one looking only to the personal accumulation of dollars and cents.

The Dean of the dental department as well as Dr. Harris will probably be heard from, and I am sure in a satisfactory and conclusive way. Had not my motive been misjudged, my intentions corrupted, and so personal an attack made upon me and others, I should not have been obliged to write such a reply as this. I sincerely regret the necessity, but never stand back when I should go forward. B. MERRILL HOPKINSON, D. D. S.,

BALTIMORE, July 7th, 1884. No. 58 Saratoga Street.

NOTE.—Owing to an error in the "making up" of the first form, the replies of Drs. Gorgas and Hopkinson were made to change places. The latter was first received, and should have been placed first.—EDITOR.

